# Selective *in-vitro* Enzymes’ Inhibitory Activities of Fingerprints Compounds of Salvia Species and Molecular Docking Simulations

**DOI:** 10.22037/ijpr.2020.112498.13801

**Published:** 2020

**Authors:** Safak Ozhan Kocakaya, Abdulselam Ertas, Ismail Yener, Bahadir Ercan, Elif Varhan Oral, Mehmet Akdeniz, Erhan Kaplaner, Gulacti Topcu, Ufuk Kolak

**Affiliations:** a *Department of Organic Chemistry, Faculty of Science, Dicle University, 21280 Diyarbakir, Turkey. *; b *Department of Pharmacognosy, Faculty of Pharmacy, Dicle University, 21280 Diyarbakir, Turkey. *; c *Department of Analytical Chemistry, Faculty of Pharmacy, Dicle University, 21280 Diyarbakir, Turkey. *; d *Department of Biochemistry, Faculty of Medicine, Girne American University, 99320 Girne, Turkish Republic of Northern Cyprus.*; e *The Council of Forensic Medicine, Ministry of Justice, 21100 Diyarbakir, Turkey. *; f *Deparment of Chemistry, Faculty of Science, Muğla Sıtkı Koçman University, 48121 Mugla, Turkey. *; g *Department of Pharmacognosy and Phytochemistry, Bezmialem Vakif University, 34093 Istanbul, Turkey. *; h *Department of General and Analytical Chemistry, Faculty of Pharmacy, Istanbul University, 34116 Istanbul, Turkey.*

**Keywords:** Salvia, Enzyme inhibition, Molecular Docking, In silico, Carnosol, Salvianolic acid

## Abstract

Recently Nutrition and Food Chemistry researches have been focused on plants and their products or their secondary metabolites having anti-alzheimer, anti-cancer, anti-aging, and antioxidant properties. Among these plants *Salvia* L. (Lamiaceae) species come into prominence with their booster effects due to high antioxidant contents, which have over 900 species in the world and 98 in Turkey. Some *Salvia *species are already in use as herbal treatment of vessel stiffness, Dementia like problems and cancer. Recently some species of *Salvia* are of extensive research topic. In this study, inhibitory potentials of secondary metabolites, rosmarinic acid, salvigenin, salvianolic acid A and B, tanshinone I and IIA, cyrtotanshinone, dihydrotanshinone I, carnosic acid, carnosol, and danshensu sodium salt were investigated against acetylcholinesterase, butyrylcholinesterase, urease and tyrosinase enzymes both *in-vitro* and *in slico* in detail. Elevated inhibitory effects on acetyl- and butyryl-cholinesterase of dihydrotanshinone I (IC_50_: 1.50 ± 0.02 and 0.50 ± 0.01 µg/mL, respectively), carnasol (IC_50_: 11.15 ± 0.05 ve 3.92 ± 0.03 µg/mL) and carnosic acid (IC_50_: 31.83 ± 0.65 ve 4.12±0.04 µg/mL) were observed. Furthermore, all other secondary metabolites were active against butyrylcholinesterase. Anti-urease (42.41 ± 0.85%) and anti-tyrosinase (39.82 ± 1.16%) activities of tanshinone I were also observed. Potential inhibitory effects of these molecules on target proteins were investigated using DOCK and molecular dynamics calculations. Dock score analysis and Lipinski parameters were demonstrated that these ligands are potential inhibitors against relevant enzymes. Our findings suggest that *Salvia* species can be utilized as a ptential source of anti-alzheimer active compounds for designing novel products.

## Introduction

Depending on the life expectancy and the aging of the world’s population, various health problems come into prominence both socially and economically. Recently, a single drug group, acetyl-cholinesterase (AChE) inhibitors have been used in the treatment of Alzheimer’s disease. Nonetheless, due to the singularity in treating mild to moderate Alzheimer’s disease with AChE inhibitors and their side effects, make it necessary to find new anti-Alzheimer’s drugs. Nowadays, increase in chronic diseases and cancers besides the extension of human life has triggered the interest in natural drugs (1). 

Tyrosinase is an enzyme coded by TYR gene and found in melanosomes of eye, skin, and hair. Abnormal melanin production (hyperpigmentation) is a common serious aesthetic problem of mid-age and elderly people (2-4). Generally, it is a sign of aging and in most cultures decreases life quality socially (5). It has been proposed that using sulphite compounds for slowing down the progress of melanosis is an effective and practical method. However, sulfur dioxide accumulation can cause health problems especially in asthma patients (2-4). 

Urease catalyses the hydrolysis of urea into ammonia and carbamic acid and is found in plants, algea, some fungi, and bacteria species. Produced ammonia is used by microorganisms in soil and plants as an nitrogen source. Urease in plants most probably participate in nitrogen transport pathways and functions as a defence protein. In humans and animals urease has role in urinary and gastrointestinal track infections and virulence factor that causes urinary tract Stones (6). 

Recently researches have focused on enzyme inhibitory potentials of secondary metabolites and/or herbal metabolites (7-13). Besides, there are ongoing docking studies of these metabolites (14, 15). Likewise seeking for a natural inhibitor as an anti-Alzheimer agent is also ongoing (16, 17). 

There are over 900 *Salvia *L. species (Lamiaceae) worldwide and 98 in Turkey naturally growing where 53 out of 98 are endemic (18). Many *Salvia *species are of homeopathic importance due to beneficial terpenoid and flavonoid contents (19, 20). Rosmarinic acid, carnosol and carnosic acids are some of the biologically active polyphenolic compounds of Lamiaceae family and *Salvia* species. Nowadays, it has been thought that polyphenolic compounds will be an phytochemical alternative to anticancer medication which have serious side effects.

The development of novel drugs is certainly one of the most demanding task of Today’s science that is provoked by the collective endeavor of the pharmaceutical industry, biotechnological companies, regulatory authorities, academic researchers, and the other private and public sectors. The augmantation of the new drugs is a very intricate and challenging interdisiplinary practice. In modern molecular drug designing, molecular docking is very succesfully used for insightful drug-receptor interaction and it provides valuable information about receptor interactions and is repeatedly used to predict the binding orientations of ligands to their protein targets in order to evaluate the binding affinity, stability, and activity of the drug candidate. 

**Figure 1:**
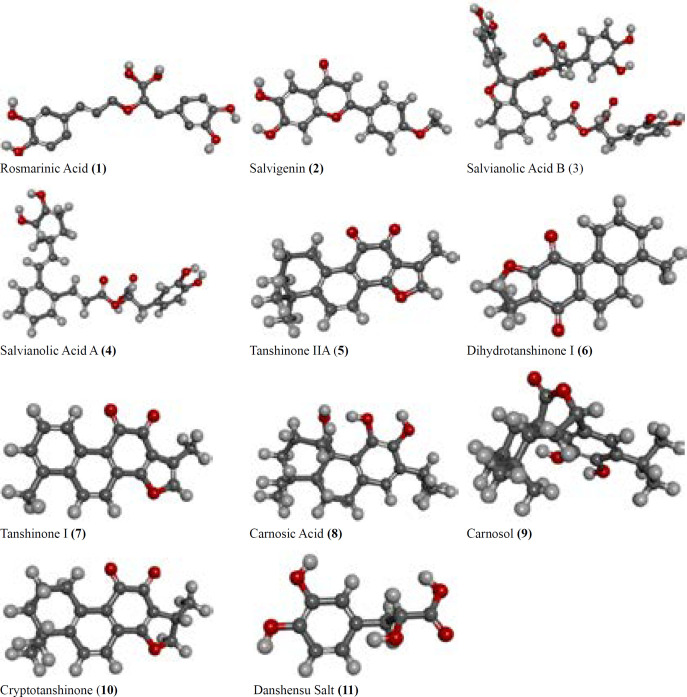
The structures of potent inhibitors

Consequently, we carried out the present study in order to test eleven phenolic, phenolic diterpenoid, and nor-abietane compounds 5-11 (Commercial available) (tanshinone IIA, dihydrotanshinone I, tanshinone I, carnosic acid, carnosol, cryptotanshinone, and danshensu salt) and 1-4 (rosmarinic acid, salvigenin, salvianolic acid B, salvianolic acid A) isolated by our group from *Salvia cerino-pruinosa *Rech., against AChE and BChE as well as urease and tyrosinase *in vitro* (Figure 1) (21). Also, the active compounds were preceeded to further studies using *in silico *experiments and molecular docking methods.

## Experimental

*Tested compounds*

Commercially available compounds 5-11 (5: Tanshinone IIA ≥97%, 6: Dihydrotanshinone I ≥98%, 7: Tanshinone I ≥97%, 8: Carnosic Acid ≥97%, 9: Carnosol ≥98%, 10: Cryptotanshinone ≥97%, 11: Danshensu Salt ≥98%) were purchased in pure form from Sigma-Aldrich Co, while compounds 1-4 (1: Rosmarinic acid, 2: Salvigenin, 3: Salvianolic acid B, 4: Salvianolic acid A) were earlier isolated by our group from *Salvia cerino-pruinosa *(21) Figure 1. 

*Chemicals*

5,5-Dithiobis-(2-nitrobenzoic acid) (DTNB), acetylcholinesterase (AChE), butyrylcholinesterase (BChE), α-tocopherol, potassium peroxodisulfate (K_2_S_2_O_8_), dichloromethane, ethanol, and galanthamine hydrobromide were purchased from Sigma-Aldrich (Steinheim, Germany), acetylthiocholine iodide reagent from Applichem (Germany), butyrylcholine iodide (Fluka), sodium carbonate, ammonium acetate, sodium hydrogen phosphate, and sodium dihydrogen phosphate from Riedel-de-Haen (Germany), urease (from Canavalia ensiformis (Jack bean) Type III, powder, 15,000-50,000 units/g solid), Phenol (≥99%), Urea (≥99.5%) and Kojic acid (analytical standard) from Sigma (Germany), tyrosinase (from mushroom lyophilized powder, ≥1000 unit/mg solid) and 3,4-Dihydroxy-L-phenylalanine (≥98%) from Sigma (Germany), thiourea (≥99.0%) from Merck (Germany).

*Biological activities and molecular modeling of the compounds*

Details of all biological activity and molecular modeling studies are given as supplementary material. The enzyme activities of the extracts were studied by observing urease, tyrosinase acetyl- and butyryl-cholinesterase inhibition activities (22-25). The ligands were then applied with partial atomic charges derived by ﬁtting Antechamber obtained by electronic structure calculation module in AMBER and assigned the AM1-BCC charges (26-29). 

## Result and Discussion

*Anticholinesterase inhibitory activity*

Dementia is a general term for a decline in mental ability severe enough to interfere with daily life. Alzheimer’s disease (AD) is the most common dementia form. It is a chronic neurodegenerative disease characterized with progressive memory loss, loss of ability to carry on even a simple conversation, and respond to their environment (30, 31). 

In 1906, a German clinical psychiatrist and neuroanatomist Alois Alzheimer reported a peculiar severe disease process of the cerebral cortex characterized by distinctive plaques and neurofibrillary tangles in the brain in a 50-year-old woman (32). Alzheimer’s disease is a serious problem and there is no effective treatment against it still after more than 100 years. 

Astrocytes and microganglions are the major cells that participate in immune/inflammatory response in AD. The patients with AD have more astrocytes and they become activated to secrete prostaglandins. Common intercellular structures in AD is amyloid plaques formed by amyloid beta (Aß) peptides. There are two types of Aß peptides; Aß-42 and Aß-40. These two forms a fibrillar structure which in turn forms amyloid plaques. Resident immune cells of brain, microglia cells surround these newly formed amyloid plaques and releases free radicals. Amyloid plaques triggers oxidative stress and neurofibrillary tangles formation. Microtubule related Tau proteins conserve microtubule integrity. But in AD these proteins are hyperphosphorilated and the binding capacity to microtubules decreases and accumulate in neurofibrillary tangles (29-31, 33). Important studies have been carried out to decrease the progression of the disease by developing inhibitors against AD. Recently nutrition and food chemistry resaerches have focused on anti-Alzheimer, anti-cancer and antioxidant potentials of herbal products and most strikingly studied one is the genus *Salvia *L. Some of the *Salvia* species were studied intensively due to their beneficial medicinal properties (33). 

High antioxidant capacity of phenolic compounds of *Salvia* species make them a potential drug candidate for Alzheimer’s Disease. Inhibitory effects of analytical grade commercially available compounds 5-10 (tanshinone IIA, dihydrotanshinone I, tanshinone I, carnosic acid, carnosol, cryptotanshinone, danshensu salt) and compunds 1-4 (rosmarinic acid, salvigenin, salvianolic acid B and salvianolic acid A), which were previously isolated by our group from *Salvia cerino-pruinosa, *were determined against anticholinesterase (Table 1). All compounds were assayed* in-vitro *against AChE and BChE at 100-0.1 µg/mL. All compounds have inhibitory effects against AChE and BChE. Dihydrotanshinone I has the highest inhibitory effect against AChE and BChE (IC_50_: 1.50 ± 0.02 µg/mL and IC_50_: 0.50 ± 0.01 µg/mL, respectively) and more active than the standart compound, galanthamine.

There are some products on the market rich in rosmarinic acid, carnosol, and carnosic acid. These compounds are used with antioxidant supplements for improvement in motivation, keeping fit and preserving Long-term memory. In our study, carnosol and carnosic acid have greatly inhibit AChE and BChE (IC_50_: 11.15 ± 0.05 ve 31.83 ± 0.65 µg/mL for AChE and IC_50_: 4.12±0.04 ve 3.92 ± 0,03 µg/mL for BChE respectively). Major secondary metabolites of *Salvia* species, rosmarinic acid, salvigenin, salvianolic acid A and B have inhibitory effects against BChE (IC_50_: 12.76 ± 0.12, 11.46 ± 0.16, 48.32 ± 0.42 ve 10.47 ± 0.10 µg/mL, respectively). Salvianolic acid A has a great inhibitory effect against AChE (IC_50_: 23.04 ± 0.16 µg/mL)

There are several studies on *Salvia* species naturally grown in Turkey. Orhan *et al.* (34). showed that 14 *Salvia* species have great antioxidant and anti-cholinesterase activity (34). Demirezer *et al.* (35) studied 3 *Salvia *species and reported that all three species have antioxidant and anti-choliesterase activity (35). It is also known that in Turkish history especially in Ottoman period *Salvia* species were used for the treatment of amnesia disease (33-38). Anti-Alzheimer activity of *Salvia* species can be refered to the secondary metabolites such as rosmarinic acid, carnosol, carnosic acid, salvigenin and salvianolic acid where last two are unique *Salvia* species grown in Turkey. Ramirez *et al.* (39) reported that carnosol has a better inhibitory effect (IC_50_: 5.1 μM) than a standart BChE inhibitor donezepil (IC_50_: 8.568 μM) against BChE (39). Szwajgier (40) determined inhitory activity of carnosic acid against both BChE and AChE (40). To date as far as we know there are no study on the anti-cholinesterase activites of Salvianolic acids A and B.

Tanshinones isolated from *S. miltiorrhiza* together with phenolic acids have shown protective effect against *β-*amyloid-induced cytotoxicity and acted as inhibitors of AChE, probably with dual mechanism of action (41). Tanshinone I, tanshinone IIA, cryptotanshinone, and 15,16-dihydrotanshinone were demonstrated to reverse scopolamine induced cognitive impairments using passive avoidance task test in mice by Kim *et al.* (42). Our current investigation revealed significant inhibitory activity of the tested compounds against BChE, and mostly a weak inhibition against AChE. Among them, the most potent compound against AChE was found to be dihydrotanshinone I with 64.54±0.36% of inhibition (IC_50_: 1.50 ± 0.02 µg/mL) having affinity toward BChE (87.19 ± 0.23%, IC_50_: 0.50± 0.01 µg/mL) which was even higher than that of galanthamine (67.52 ± 0.41%, IC_50_: 6.19 ± 0.12 µg/mL). Our results are parallel to the results of Senol et al (2017). Besides that IC_50 _value of dihydrotanshinone I was 1.71 µg/mL where we found is 0.50 µg/mL. Additionally, in that study inhibitory acitivities of tanshinone IIA, cryptotanshinone, dihydrotanshinone I, tanshinone I, and rosmarinic acid against AChE had been carried out but no molecular modelling studies had been performed. 

**Figure 2.**
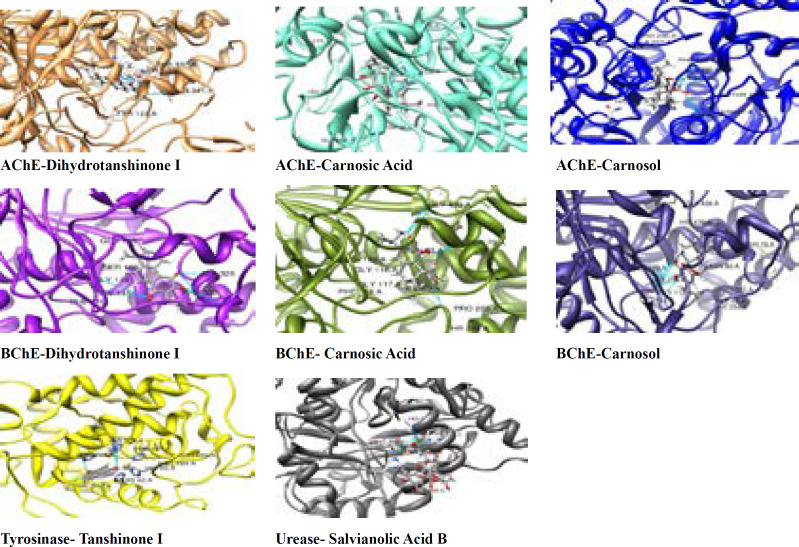
Docking results of ligands in catalytic pocket of enzymes

**Figure 3:**
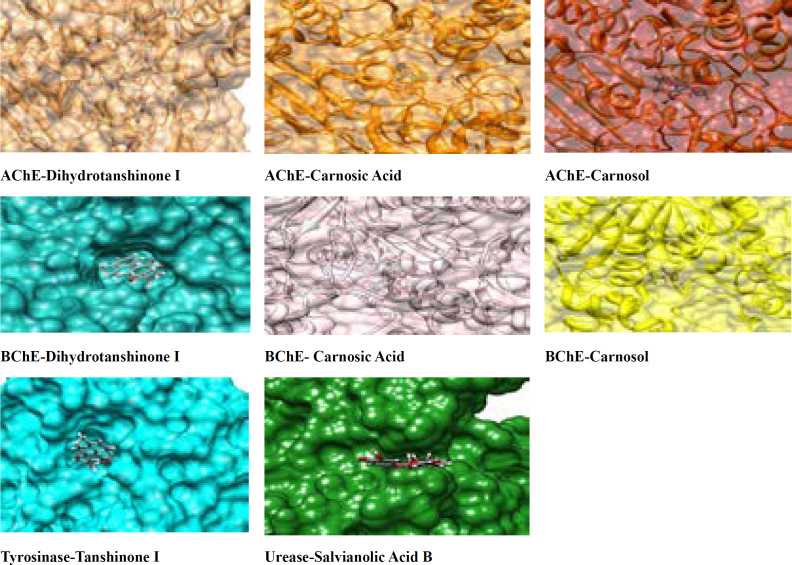
Surface representations of the active sites of enzymes with the bound ligands. The wide opening of the binding site pocket allows compounds to adopt flexible conformation in this area

**Figure 4:**
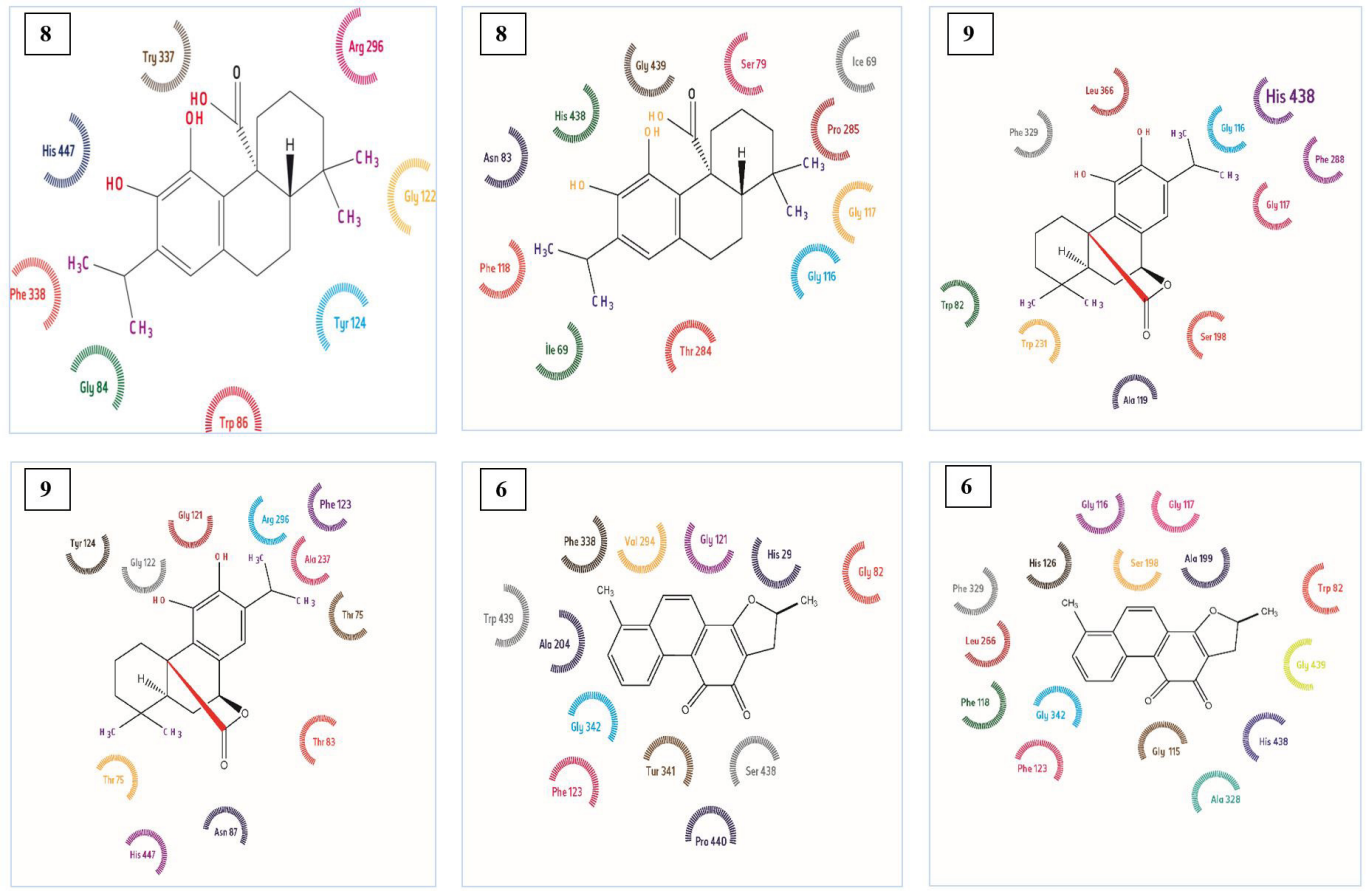
Binding mode of complex Carnosic Acid (8), Carnosol (9) and Dihydrotanshinone I (6) with AChE and BuChE in 2 D representation

Enzyme-inhibitor interactions were assessed with the help of docking calculations where binding free energy was recorded at each possible position. Molecular Docking results are shown in Table 2. The energy of complexation was observed in range from -15.94 kcal/mol to -52.44 kcal/mol, respectively. The calculated interaction of Dihydrotanshinon I with the active site of AChE displayed that entrenched in a remarkable group of amino acid with aromatic ring including Trp 86, Tyr 337, and Tyr 124. (Figure 1-4) The compound was dock with AChE with its active site available whereas ligand located parallel to Trp 86 and Tyr 337 constitude π-π stacking. Hidrogen attached to O and atom making hydrogen bonding with –OH group of His 447, Phe 338 and another hydrogen bond were also formed with –OH group of Tyr 337 and Gly 122. Similarly, Carnosic acid and Carnosol molecules also making hydrogen bonding with –OH group via His 447 and Tyr337 within the active site of AChE. (Figure 1-4)

The molecular docking experiments on BChE active site indicate well established polar interactions and Hydrogen bondings. Observed energy and complexlation range are from -35.22 kcal/mol to -60.27 kcal/mol, respectively. In Table 2 the type of binding interaction between inhibitor molecules and BChE was different from interaction among inhibitors and AChE owing to a difference of amino acid chain in active site of BChE. 

The type of binding interaction between inhibitor molecules and BChE was different from interaction between inhibitors and AChE due to a difference in amino acid residue in active site of BChE.

Aromatic π-π stacking accured between Trp 231, Trp 82, and Phe 118. Molecular docking has also releaved some other important interactions such as hydrogen bonding with Gly116, Gly117, His 438, and Thr 284. (Figure 4) 

*Tyrosinase and urease inhibitory activities*

Antiurease and anti-tyrosinase activities of 11 compounds were determined and results are given as µg thiourea or kojic acid activity per mg compound (Table 1). Salvianolic acid B showed the best antiurease activity among the tested compounds (192.26 ± 0.21 µg thiourea Activity/mg compound). As for Anti-tyrosinase activity tanshinone I showed better activity with a 372.86 ± 2.47 µg kojic acid activity/mg compound.

There are few studies on anti-tyrosinase activity of the compounds obtained from *Salvia* species in the literature (43, 44). Studies mostly focused on various extracts of *Salvia* species rather than their compounds. Zengin *et al.* (43) determined that the water extracts of *S. sclarea* have reasonable antityrosinase activity. Our study indicated that except tanshinone I none of the other compounds showed a reasonable antityrosinase activity. 

**Figure 5.**
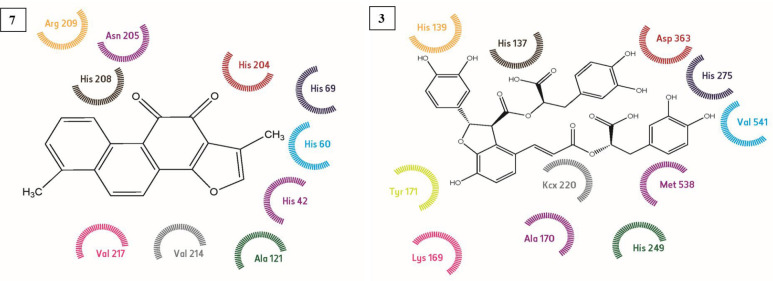
Binding mode of complex Tanshinone I (7) with Tyrosinase and complex Salvianolic acid B (3) with urease in 2 D representation

Using the known cristallograpic structure of tyrosinase of 5i38.pdb, we performed molecular dynamic and docking methods. As shown in Figure 2-3 the docked orientations showed that all ligands were located in the hydrophobic binding pocket. The calculated energy of the ligands range from-14.44 kcal/mol to -30.99 kcal/mol, respectively. All docked ligands were found to interact between an oxygen atom of the ligands and histidine residue within 4 Å. In the binding pocket, common protein-ligand interactions were formed between all docked ligands and Asn 205, His 204, His 208, His 69, His 60, His 62, Val 214, Val 217, and Val283. In order to explain the binding of these compounds, docking simulation has also releaved some other important interactions such as hydrogen bonding with Val 214, Val 217, Ala 221 (Figure 2-3, 5). The specific interaction between tanshinone I and tyrosinase predicted the docked structure in the active site of enzyme shown in Figure 5.

There are a few studies on the anti-urase activity of *Salvia *extracts and their secondary metabolites in the literature. Huang *et al*. (45) investigated the effects of water extract of *S. plebeia* on intestinal movements of the rats (45) and found that sufficient enough extract (0.5/100 g diet) can protect intestine via decreasing exposure of toxic compounds on intestinal mucosa. In our study all compounds except for salvianolic acid B (Inhibisyon%: 49.83 ± 0.27) and tanshinone I (Inhibisyon%: 42.41 ± 0.85) showed low anti-urease activity.

The ligands bind to urease as inferred by their minimum energy values that range from-8.14 kcal/mol to-33.43 kcal/mol, respectively. This is a good agreement with experimentally observed IC_50_ values for these compounds (Table 1). Figure 2-3 shows the best conformation of salvianolic acid B into binding pocket of urease. Hydrogen bond acts as imported factor for contributing in protein-ligand stability. They generally posses 3 A between the H-donor and H acceptor atoms. Figure 1-2, 5 displays hydrogen bond interactions between protein and ligands. Likewise, hydrogen bonding, the van der Waals interaction also play a vital role in the protein stability. The amino acid residues that are involved in van der Waals interactions are all hydrophobic in nature as expected. The current study reveals that Ala170, Val 541, Met 538 participiate in van der Waals interactions with ligands. The binding model of the ligands with urease indicate Ni^+2^, Ni^+2^ KCX 220, ASP 363, Lys 169, His 139, Met 538, His 275, His 249, His 137, His 139 and Tyr171 as major residues involved (Figure 5).

Calculated Lipinski values generally supported Dock score results and showed that especially Dihydrotanshinone I, Carnosic acid, and Carnosol molecules were considerable (46) (Table 3).

**Table 1:** Inhibition %, IC_50_ values and Equivalents of the Compounds (1-11) against AChE, BChE, Urease and Tyrosinase^a^.

		**Inhibition (%)** ^b^				**IC**_50_ **(µg/mL)**		**Equivalent (µg kojic acid or** **thiourea activity/ mg compound)**	
**No**	**Compounds **	**AChE**	**BchE**	**Urease**	**Tyrosinase**	**AChE**	**BChE**	**Urease**	**Tyrosinase**
1	Rosmarinic Acid	20.18±0.21	42.76±0.32	N.A.^c^	12.11±0.02	>100	12.76±0.12	N.A.	105.58±0.18
2	Salvigenin	22.72±0.14	43.13±0.21	N.A.	N.A.	>100	11.46±0.16	N.A.	N.A.
3	Salvianolic Acid B	10.11±0.08	49.83±0.27	45.52±0.68	N.A.	>100	10.47±0.10	192.26±0.21	N.A.
4	Salvianolic Acid A	38.82±0.12	27.49±0.08	N.A.	N.A.	23.04±0.16	48.32±0.42	N.A.	N.A
5	Tanshinone IIA	32.34±0.44	66.26±1.18	14.26±0.42	N.A.	>100	1.12±0.02	16.12±0.01	N.A.
6	Dihydrotanshinone I	64.54±0.36	87.19±0.23	N.A.	N.A.	1.50±0.02	0.50±0.01	N.A.	N.A.
7	Tanshinone I	31.29±0.72	33.01±0.86	42.41±0.85	39.82±1.16	38.12±0.62	27.67±0.04	174.73±0.19	372.86±2.47
8	Carnosic Acid	37.99±0.96	58.48±1.18	22.42±0.15	5.92±0.06	31.83±0.65	4.12±0.04	62.12±0.07	45.93±0.71
9	Carnosol	47.60±023	57.99±0.15	16.12±0.42	N.A.	11.15±0.05	3.92±0,03	26.60±0.03	N.A.
10	Cryptotanshinone	17.46±0.11	35.16±0.27	29.69±1.16	N.A.	>100	28.41±0.65	103.05±0.11	N.A.
11	Danshensu Salt	N.A.	35.37±0.13	N.A.	6.20±0.08	N.A.	24.18±0.42	N.A.	48.67±0.23
	Galanthamine^d^	76.08 ± 0.39	67.52 ± 0.41	-	-	5.13±0.02	8.19±0.12	-	-
	Kojic acid^d^	-	-	-	69.07±0.38	-	-	-	-
	Thiourea^d^	-	-	75.14±1.34	-	-	-	-	-

^a^Values expressed are means ± S.D. of three parallel measurements and values were calculated according to negative control. were significantly different (*p* < 0.05)

^b^10µg/mL

^c^Not active

^d^Standart compounds

**Table 2:** Calculated thermodynamic parameters for complexation of ligands by docking method

**No**	**Compounds**	**AChE**			**BchE**			**Urease**			**Tyrosinase**		
		VdW	es	DockS	VdW	es	DockS	VdW	es	DockS	VdW	es	DockS
1	Rosmarinic Acid	-40.33	-1.49	-41.82	-38.98	-5.17	-44.157	-7.23	-0.85	-8.14	-29.23	-0.29	-29.52
2	Salvigenin	-29.39	-1.75	31.14	-47.09	-13.68	-60.27	-15.25	1.18	16.43	16.13	-1.12	-17.25
3	Salvianolic Acid B	-13.51	-2.43	-15.94	-3.10	-34.76	-37.86	-28.17	-5.26	-33.43	-13.54	-2.35	-15.89
4	Salvianolic Acid A	-12.98	-28.05	-41.48	-32.04	-3.18	-35.22	-12.92	-1.65	-14.57	13.08	1.36	-14.44
5	Tanshinone IIA	-18.68	-1.56	-20.24	-55.67	-7.78	-63.45	-17.77	-2.41	-20.18	-15.21	-1.23	-16.44
6	Dihydrotanshinone I	-41.58	-10.86	-52.44	-54.96	-10.57	-65.53	-13.51	2.01	-15.52	-17.20	-0.85	-18.05
7	Tanshinone I	-28.80	-1.30	-30.11	-33.91	-2.16	-36.-1	-28.96	-1.18	-30.5	-30.79	-0.20	-30.99
8	Carnosic Acid	-20.04	-2.03	-22.07	-40.05	-2.53	-42.59	-25.47	-0.35	-25.83	-18.90	-1.21	-20.11
9	Carnosol	-9.02	-33.12	-42.14	-42.23	-1.98	-44.18	-18.15	-2.11	20.26	15.13	1.09	16.22
10	Cryptotanshinone	-5.18	-21.35	-26.53	-35.10	-2.71	-37.01	-26.83	-2.13	-28.96	18.02	-0.25	-18.27
11	Danshensu Salt	-2.99	-10.96	-13.95	-29.63	-10.01	-39.64	-16.02	-.90	-16.21	17.01	-2.31	-19.32
	Galanthamine	-66.53	-11.68	-78.21	-63.05	-7.14	-71.19						
	Kojic Acid										-69.19	-10.97	80.16
	Thiourea							-55.15	-4.18	-59.33			

**Table 3:** Calculated Lipinski parameters for complexation of compounds 1-11 by Molinspiration method

**No**	**Compounds**	**miLogP**	**Mw**	**nON**	**nOHNH**	**nRotb**
1	Rosmarinic Acid	-1.09	359.31	8	4	7
2	Salvigenin	3.23	329.32	6	1	4
3	Salvianolic Acid B	1.89	718.62	16	9	14
4	Salvianolic Acid A	3.01	494.45	10	7	9
5	Tanshinone IIA	4.16	294.35	3	0	0
6	Dihydrotanshinone I	2.92	280.32	3	0	0
7	Tanshinone I	3.83	276.29	3	0	0
8	Carnosic Acid	4.30	338.49	4	3	2
9	Carnosol	5.22	346.47	4	2	3
10	Cryptotanshinone	3.25	298.38	3	0	0
11	Danshensu Salt	-2.96	197.17	5	3	3


## Conclusion

In the current study, eleven polyphenolic derivatives were tested for their *in-vitro* effects against acetyl- and butyryl-cholinesterase, urease and tyrosinase enzymes and the promising inhibitors were revealed to be salvianolic acid A**, **dihydrotanshinone I, tanshinone I, carnosic acid, carnosol, which were further proceeded to in silico studies using molecular docking experiments. According to our findings, all of the tested compounds could be good precursor models for BChE-inhibiting molecules and among them, dihydrotanshinone I, carnosic acid, and carnosol in particular could be more promising since it can display dual inhibition on AChE and BChE enzymes effectively. Moreover, our *in silico* data well-matched with the *in-vitro* out comes of this study. As a conclusion specific compounds to *Salvia *species have lower inhibitory effects on urease and tyrosinase but have potential inhibitor effects on AChE and BChE enzymes. 
